# Validation of low-cost reflectometer to identify phytochemical accumulation in food crops

**DOI:** 10.1038/s41598-024-52713-0

**Published:** 2024-01-30

**Authors:** Carl L. Rosier, Dan Kittredge, Barbara Nainiger, Octavio Duarte, Greg Austic, Dan TerAvest

**Affiliations:** 1 Basil’s Harvest, Chicago, IL 60606 USA; 2 The Bionutrient Institute, Barre, MA 01005 USA; 3 OurSci, LLC, Ann Arbor, MI 48103 USA

**Keywords:** Biological techniques, Plant sciences

## Abstract

Diets consisting of greater quantity/diversity of phytochemicals are correlated with reduced risk of disease. This understanding guides policy development increasing awareness of the importance of consuming fruits, grains, and vegetables. Enacted policies presume uniform concentrations of phytochemicals across crop varieties regardless of production/harvesting methods. A growing body of research suggests that concentrations of phytochemicals can fluctuate within crop varieties. Improved awareness of how cropping practices influence phytochemical concentrations are required, guiding policy development improving human health. Reliable, inexpensive laboratory equipment represents one of several barriers limiting further study of the complex interactions influencing crop phytochemical accumulation. Addressing this limitation our study validated the capacity of a low-cost Reflectometer ($500) to measure phytochemical content in selected crops, against a commercial grade laboratory spectrophotometer. Our correlation results ranged from r^2^ = 0.81 for protein in wheat and oats to r^2^ = 0.99 for polyphenol content in lettuce in both the Reflectometer and laboratory spectrophotometer assessment, suggesting the Reflectometer provides an accurate accounting of phytochemical content within evaluated crops. Repeatability evaluation demonstrated good reproducibility of the Reflectometer to assess crop phytochemical content. Additionally, we confirmed large variation in phytochemical content within specific crop varieties, suggesting that cultivar is but one of multiple drivers of phytochemical accumulation. Our findings indicate dramatic nutrient variations could exist across the food supply, a point whose implications are not well understood. Future studies should investigate the interactions between crop phytochemical accumulation and farm management practices that influence specific soil characteristics.

## Introduction

Phytochemicals are critical in healthy plant essential functions assisting with various environmental challenges including herbivory deterrent, pollination attractants, and protection against pathogen and abiotic stressors^1^. As a result, phytochemicals are present within domesticated crops (i.e., fruits, small grains, and vegetables) providing benefits to the consumer^2^. For example, antioxidants and phenolic compounds are considered key classes of disease preventing phytochemicals due to their capacity to quench cell damaging free radicals^3^. At the opposite end of the phytochemical spectrum plant proteins play a pivotal health promoting role in the human diet^4^. Epidemiological studies suggest that diets incorporating a greater proportion and diversity of plant-based phytochemicals are significantly correlated with reduced risk of chronic diseases including cancer, cardiovascular disease, diabetes, and obesity^5^.

Despite the importance of phytochemicals to human health, multiple studies have reported decades-long declines in crop nutrient content. A clear mechanistic understanding of multiple factor interactions (i.e., crop variety, environment, and management practices) contributing to reported declines in crop mineral and phytochemical accumulation remains elusive. Mayer^6^ provided the first assessment of declines in mineral content of fruits and vegetables using historical data tables (U.K. Chemical Composition of Foods reports) spanning a 50-year period (1936–1991). They suggested that modern agricultural practices could be the mechanism behind observed declines but noted additional investigations were necessary. Utilizing a similar approach, Davis et al.^7^ evaluated crop nutrient content (43 fruits and vegetables) using historic data (USDA Food Composition Table) covering 50 years (1950–1999). Their observations indicate that changes in individual crop mineral content could not be assessed due to large variation and uncertainties in collected data, however, when data was grouped declines in both Ca (16%) and Fe (15%) were noted. Mechanistically they suggest that observed differences in nutrient content could be the result of cultivar selection for yield, crops increased synthesis of starches and carbohydrates do not occur proportionally to nutrient accumulation (i.e., dilution effect). Marles^8^ extensively reviewed previous research efforts investigating declines in crop nutrient content, they suggest that studies utilizing historic data sets are not a valid approach often providing misleading conclusions calling for side-by-side comparison studies. Perhaps a pertinent question then, is not what the average nutrient levels in crops now are in relation to where they were in the past, but what the potentials are and how can we begin to increase their concentration across the food supply. In one of a few paired studies Montgomery et al.^9^ investigated potential differences in mineral content and phytochemical accumulation across vegetable and grain crops comparing conventional and organic management. Their results suggest greater mineral content and phytochemical accumulation in crop samples organically managed compared to conventional farming methods. They attributed reported differences to greater SOM and biological diversity within organic managed soils.

To address issues of nutrient quality and human health outcomes, nationally recommended dietary (NRDs) guidelines have been developed globally by several governmental agencies and advisory committees to increase public awareness of the importance of consuming fruits, small grains, and vegetables with the intended objective of improving overall human health outcomes^10^. Currently there is a growing demand for governmental oversight to develop policies requiring crop production to focus on healthy and sustainable diets defined as providing essential minerals and vitamins coupled with minimal environmental impact^11^. Regenerative agriculture, a type of agriculture broadly defined as using practices that increase soil organic matter and soil health, has been suggested to improve nutrient quality, increase soil health, and protect ecosystem services^12^. These practices and techniques stem from indigenous wisdom and the Organic, Biodynamic, Agro-ecological, Permaculture, AMP etc. communities. The Regenerative Agriculture movement has developed a significant following and has captured the interest of consumer markets, corporations, and certification schemes^13^. Improved nutritional quality of crops is a key outcome indicator of successful regenerative agriculture^12,13^.

Due to the complex interactions between crop variety, environment, management practices, harvesting conditions/methodologies, as well as length of storage effects on phytochemical accumulation and stabilization additional studies are required to adequately understand the drivers of nutritional quality or back claims of increased nutrient quality from Regenerative Agriculture. The paucity of research efforts to date untangling factors influencing mineral and phytochemical accumulation in crops is in part due to a lack of consensus regarding standard testing methodologies and cost-effective accessible laboratory instrumentation. The combination of colorimetric assays and optical spectroscopy provides a valuable suite of analytical tools for quantifying phytochemical content. Indeed, there are many published colorimetric methods for quantifying phytochemical content however, typical spectrometers utilized for this purpose are expensive and require complex downstream data processing software and advanced technical training thus limiting their widespread application^14–16^. Development and application of inexpensive, robust reflectometers to quantify phytochemicals using colorimetric analysis could significantly lower analysis costs and scale up data collection, enabling larger scale studies within the food supply chain. In tandem with this requirement simpler data collection platforms and processing pipelines would allow improved repeatability, ensuring data comparability across large scale projects.

The overarching goal of this study is to validate the capacity of the low cost ($500) Bionutrient Meter (hereafter referred to as the Reflectometer) to assess antioxidant activity and phenolic and protein content of selected crop cultivars using colorimetric assays. After successful validation of the Reflectometer, we next investigated crop phytochemical variability focusing our assessment on cultivar variety. Our specific objectives include: (*i*) verify the capacity of the Reflectometer to accurately assess the phytochemical (i.e., antioxidant, phenolic and protein) content comparing reflectometer reflectance spectra against commercial-grade spectrometer absorbance data collected from both prepared standard curves and selected crop samples and (*ii*) assesses variation in antioxidant, phenolic, and protein content broadly, and as a function of crop variety in lettuce, carrot, oat, and wheat samples.

## Results

### Methodology validation study

**Figure 1:**
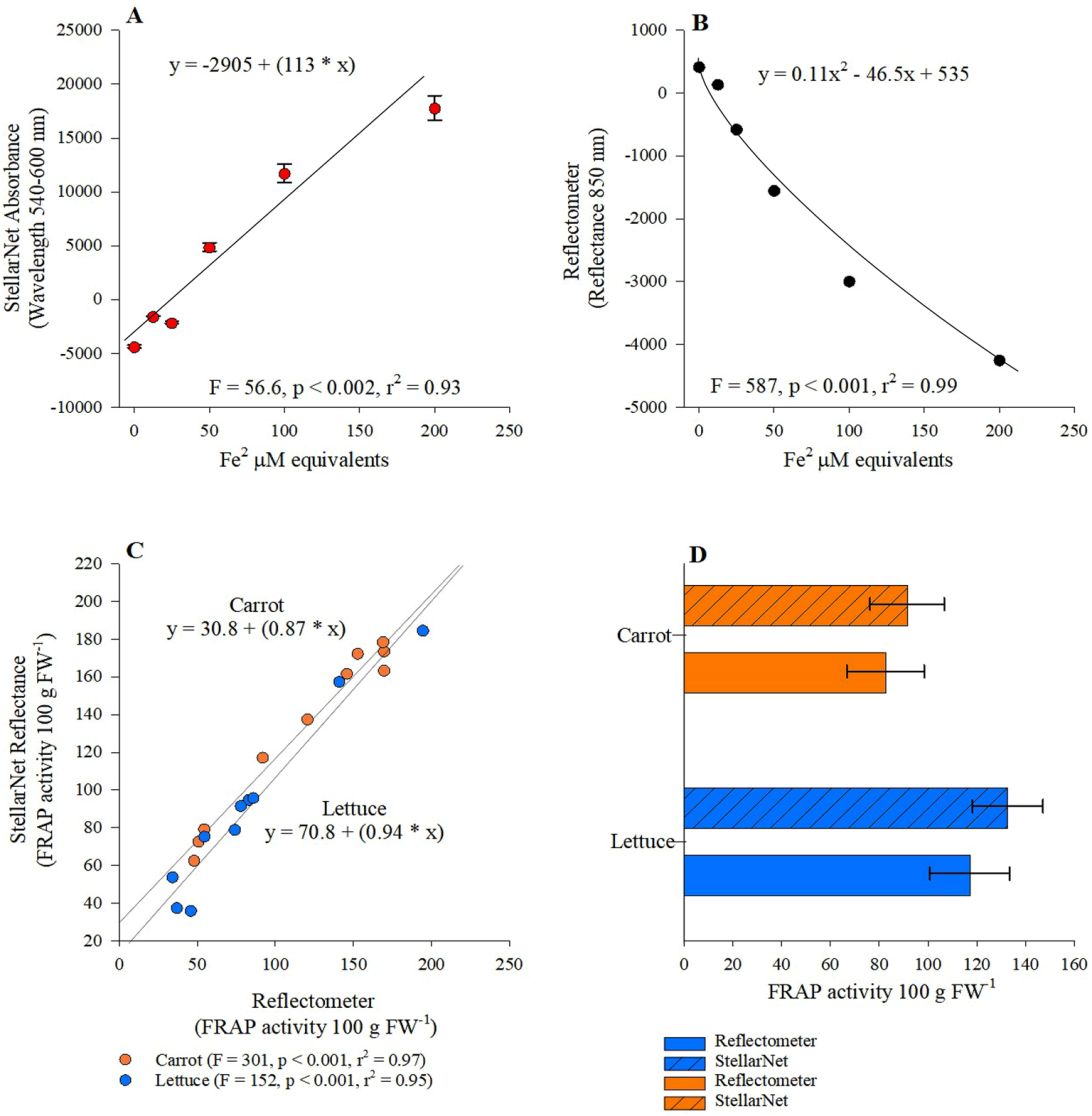
HYPERLINK "sps:id::fig1||locator::gr1||MediaObject::0"(**A**,**B**) Relationship between ferric reducing antioxidant power (FRAP) standard curve concentrations generated by increasing concentrations of ferrous chloride (FeCl^3^) yielding Fe^2^ µM equivalents and StellarNet absorbance averaged across 540–600 nm wavelength, (**B**) relationship between Reflectometer reflectance calculated at 850 nm, (**C**) relationship between Reflectometer and StellarNet absorbance assessing FRAP concentrations calculated from Lettuce (blue circles) and Carrot (orange circles) samples and (**D**) comparing carrot and lettuce FRAP concentrations measured by both Reflectometer and StellarNet spectrometer.

Our assessment of the Reflectometer’s capacity to accurately quantify concentrations of antioxidants initially involved determining reflectance output of the StellarNet spectrometer across a wavelength range of 540–600 nm using a standard curve array of 0–200 (Fe^3^ µm). Our results indicate a significant positive linear increase in reflectance values as Fe^3^ concentration increased (Fig. 1A: p < 0.002, r^2^ = 0.93). In contract we observed a significant negative non-linear relationship between Reflectometer measured reflectance and increasing Fe^3^ concentrations (Fig. 1B: p < 0.001, r^2^ = 0.99). FRAP concentrations measured by the Reflectometer maintained a good fit and significant positive linear relationship with FRAP concentrations measured via StellarNet spectrometer for both carrot (Fig. 1C: p < 0.001, r^2^ = 0.97) and lettuce samples (Fig. 1C: p < 0.001, r^2^ = 0.95). Comparison of both carrot and lettuce FRAP concentrations measured by the Reflectometer and StellarNet spectrometer suggest no significant differences between either method, in both instances the Reflectometer underestimated FRAP concentrations (Fig. 1D).

**Figure 2:**
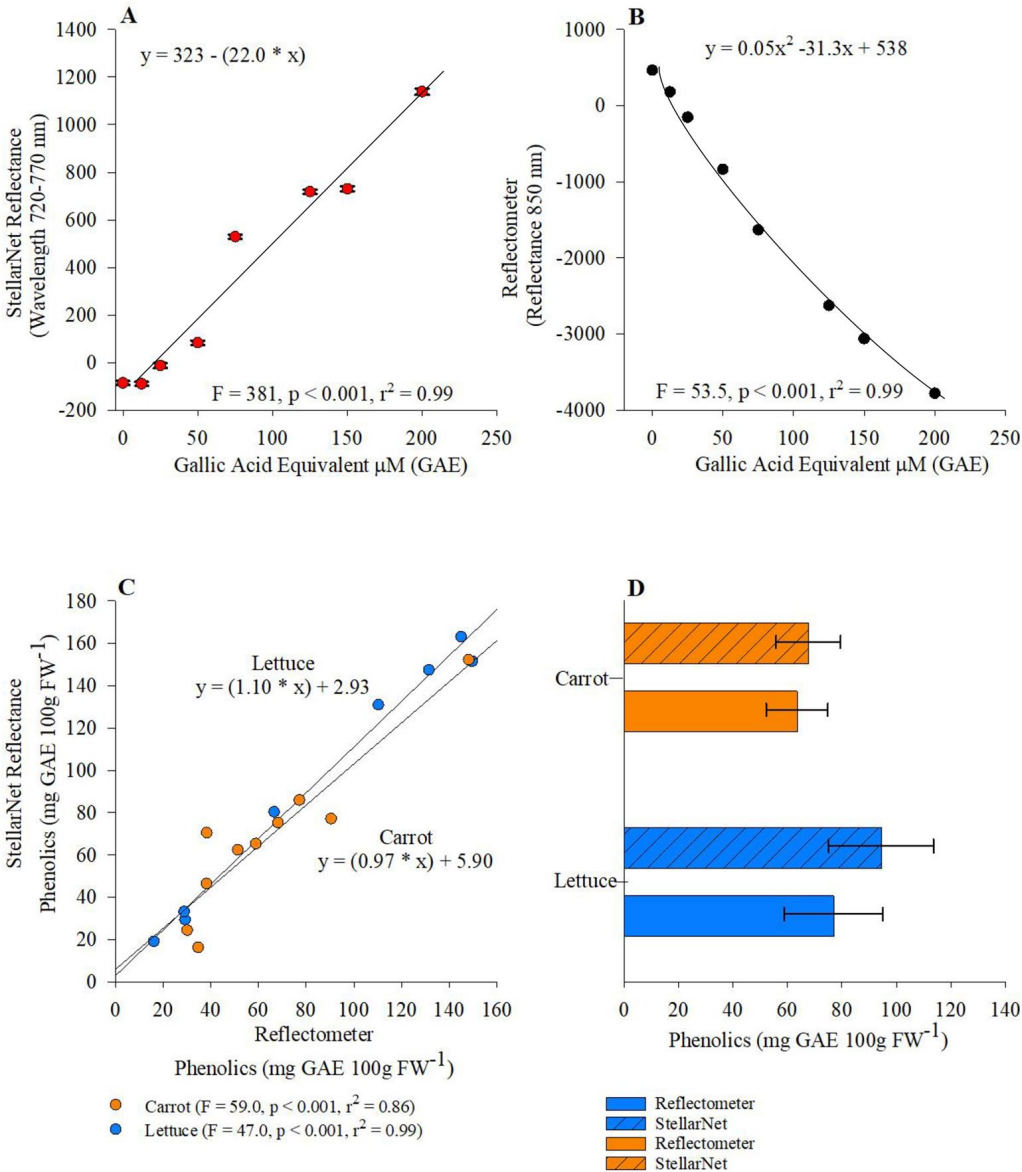
(**A**) correlation between phenolic standard curve concentrations and StellarNet absorbance averaged across 720–770 nm wavelength, (**B**) relationship between Reflectometer reflectance calculated at 850 nm, (**C**) correlation between Reflectometer and StellarNet absorbance assessing phenolic concentrations calculated from Lettuce (blue circles) and Carrot (orange circles) samples, and (**D**) carrot or lettuce samples phenolic content observed from StellarNet spectrometer and Reflectometer.

The Folin Ciocalteau spectrophotometric method was used to test the accuracy of the Reflectometer to quantify phenolic content of selected crop samples. The reflectance output of the StellarNet spectrometer was assessed using a standard curve 0–200 mg GAE with a wavelength range 720–770 nm. Our results suggest a significant linear relationship between increasing concentrations of GAE and reflectance values measured by StellarNet spectrometer (Fig. 2A: p < 0.001, r^2^ = 0.99). The Reflectometer indicates a significant non-linear negative relationship between reflectance and increasing GAE concentrations (Fig. 2B: p < 0.0001, r^2^ = 0.99). Carrot phenolic concentrations measured by the Reflectometer maintained a modest fit and significant positive linear relationship with StellarNet spectrometer reflectance values (Fig. 2C: p < 0.0001, r^2^ = 0.86), in contrast lettuce samples provided a better fit and significant linear relationship (Fig. 2C: p < 0.0001, r^2^ = 0.99). No significant differences in carrot or lettuce samples Phenolic content were observed when StellarNet spectrometer and the Reflectometer were evaluated, similar to previous findings the Reflectometer underestimates Phenolic concentrations in both samples (Fig. 2D).

**Figure 3:**
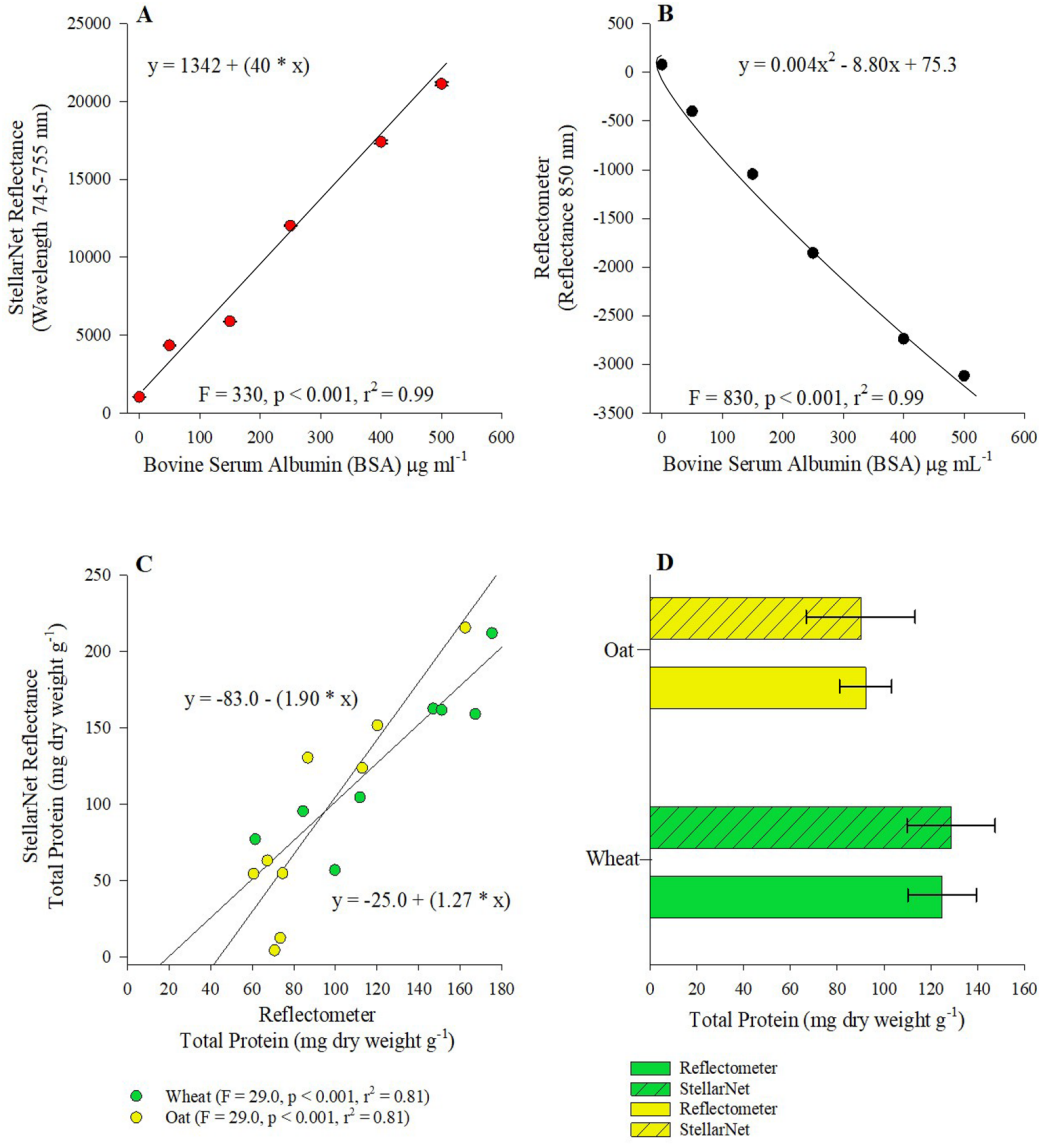
(**A**) Relationship between Lowry protein standard curve concentrations and StellarNet reflectance averaged across 745–755 nm wavelength, (**B**) relationship between Reflectometer and reflectance calculated at 850 nm, (**C**) relationship between Reflectometer and StellarNet reflectance assessing Lowry protein concentrations calculated from Wheat (green circles) and Oat (yellow circles) samples, and (**D**) Total protein content in both oat and wheat between Reflectometer and StellarNet spectrometer.

To test the efficiency of the Reflectometer to assess small grain protein concentrations we used the Lowery protein extraction method in conjunction with a standard curve composed of BSA at increasing concentrations (0–500 mg). Our results suggest a significant linear relationship between increasing concentrations of BSA 0–500 mg.at reflectance wavelength ranging from 745 to 755 nm for the StellarNet spectrometer (Fig. 3A: p < 0.001, r^2^ = 0.99). The Reflectometer provided a significant non-linear negative relationship between reflectance and increasing BSA concentrations (Fig. 3B: p < 0.001, r^2^ = 0.99). Small grain protein concentrations measured by the Reflectometer maintained a modest fit yet significant positive relationship with protein concentrations measured via StellarNet spectrometer for both oat (Fig. 3C: p < 0.001, r^2^ = 0.81) and wheat samples (Fig. 3C: p < 0.001, r^2^ = 0.81). Total protein content in both oat and wheat did not significantly differ between Reflectometer and StellarNet spectrometer (Fig. 3D).

Analyses of inter-day repeatability were carried out to evaluate the capacity of the Reflectometer to accurately determine the antioxidant, polyphenol, and protein content of standard reference samples. As noted in Table 1, the Relative Standard Deviation (RSD%) was less than 15% for all replicated tested samples spanning multiple sampling periods.

**Table 1:** Reflectometer repeatability assessment of extracted phytochemicals.

Phytochemical	Sample	Day	Mean (± sd)	RSD %
Antioxidant^a^	Squash^d^	1	50.0 (3.90)	7.80
		104	64.0 (7.20)	11.2
		141	59.0 (3.80)	6.50
		154	61.0 (3.60)	6.00
Polyphenols^b^	Squash	1	34.8 (5.00)	14.5
		182	29.2 (3.30)	11.1
		217	25.0 (2.70)	10.8
		225	29.0 (2.80)	10.1
Antioxidant	Grain^e^	1	44.0 (2.20)	5.10
		33	64.0 (7.20)	11.0
		111	64.0 (5.60)	5.60
Polyphenols	Grain	1	68.0 (2.00)	3.90
		53	38.0 (7.50)	7.50
		161	58.0 8.00)	8.00
		164	59.0 (5.30)	5.30
Protein^c^	Grain	1	73.0 (11.6)	15.0
		50	105 (6.10)	6.10
		69	107 8.80)	8.80

^a^Antioxidant concentration assessed by FRAP method.

^b^Polyphenol concentrations evaluated by Folin Ciocalteau method.

^c^Protein content determined by Lowry method.

^d^Butternut squash represents standard produce and vegetable sample.

^e^Wheat represents standard sample for both wheat and oat sample.

Sample n = 5 for each evaluation.

### Crop phytochemical variability study

#### Antioxidant capacity of differing crop varieties

**Figure 4:**
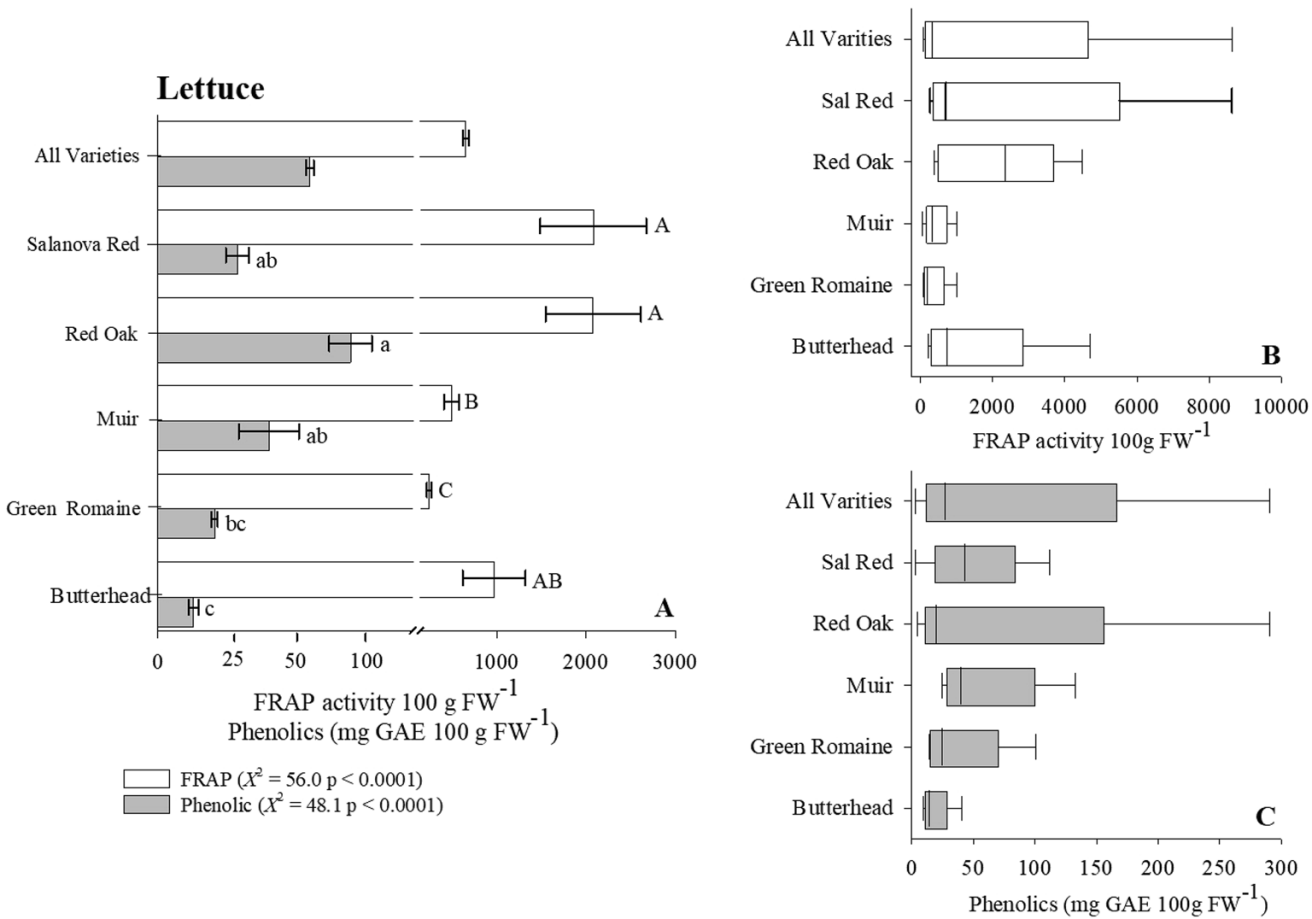
(**A**) Total ferric reducing antioxidant power (FRAP) activity and phenolic content for Lettuce. Wilcoxon One Way Analysis assessed variety differences (standard error bars with p < 0.05). Capital letters indicate significant differences between FRAP content and lowercase letters indicate significant differences between phenolic content. (**A**) x-axis break occurs between 150 and 200. (**B**,**C**) Represent Box whisker of FRAP and Phenolic distributions. Box boundaries represent quartiles, the thick line in the box is the median and the whiskers represent the samples minimum and maximum. Lettuce cultivars consist of Butterhead (n = 12), Green Romaine (n = 61), Muir (n = 8), Red Oak (n = 9), and Salvanova Red (n = 20).

**Figure 5:**
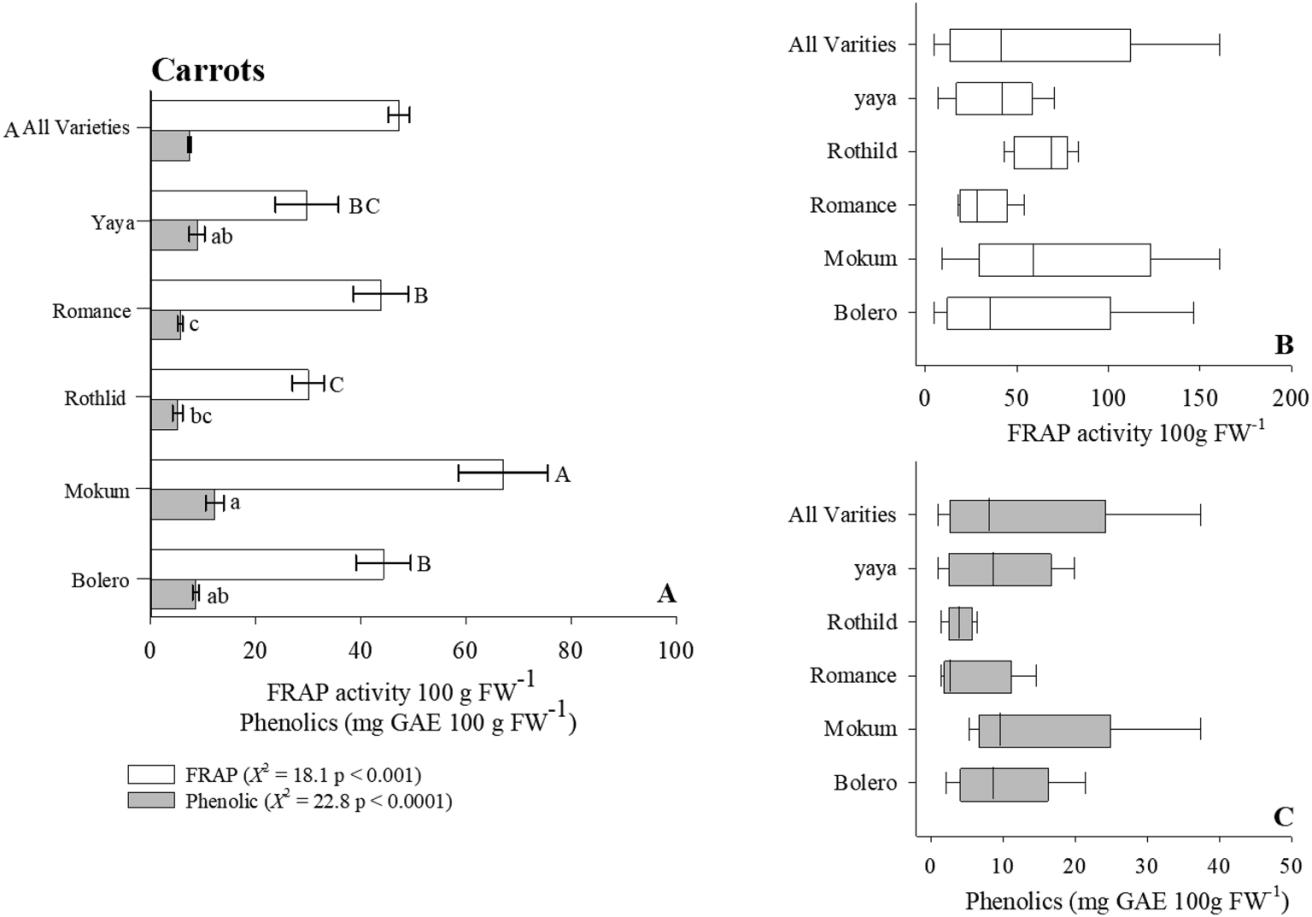
(**A**) Total ferric reducing antioxidant power (FRAP) activity and phenolic content for Carrot. Wilcoxon One Way Analysis assessed variety differences (standard error bars with P < 0.05). Capital letters indicate significant differences between FRAP content and lowercase letters indicate significant differences between phenolic content. (**A**) x-axis break occurs between 150 and 200. (**B**,**C**) Represent Box whisker of FRAP and Phenolic distributions. Box boundaries represent quartiles, the thick line in the box is the median and the whiskers represent the samples minimum and maximum. Carrot cultivars include Bolero (n = 44), Mokum (n = 21), Romance (n = 17), Rothlid (n = 7), and Yaya (n = 12).

**Figure 6:**
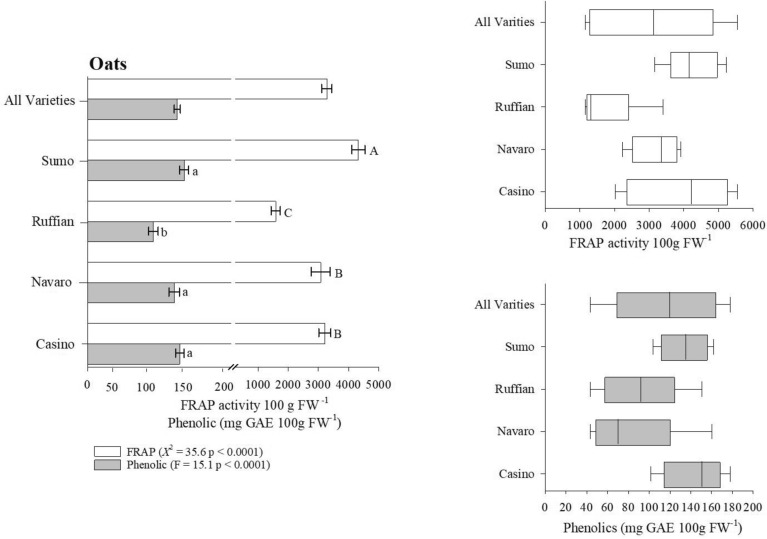
(**A**) Total ferric reducing antioxidant power (FRAP) activity and phenolic content for Oat. Wilcoxon One Way Analysis assessed variety differences for FRAP content within Oat samples. Oat phenolic assessment completed with ANOVA on log transformed data. Capital letters indicate significant differences between FRAP content, lowercase bold letters indicate significant differences between phenolic content (standard error bars with p < 0.05). (**A**) x-axis break occurs between 200 and 250. (**B**,**C**) Represent Box whisker of FRAP and phenolic distributions. Box boundaries represent quartiles, the thick line in the box is the median and the whiskers represent the samples minimum and maximum. Oat varieties consist of Casino (n = 18), Navaro (n = 9), Ruffian (n = 20), and Sumo (n = 9).

**Figure 7:**
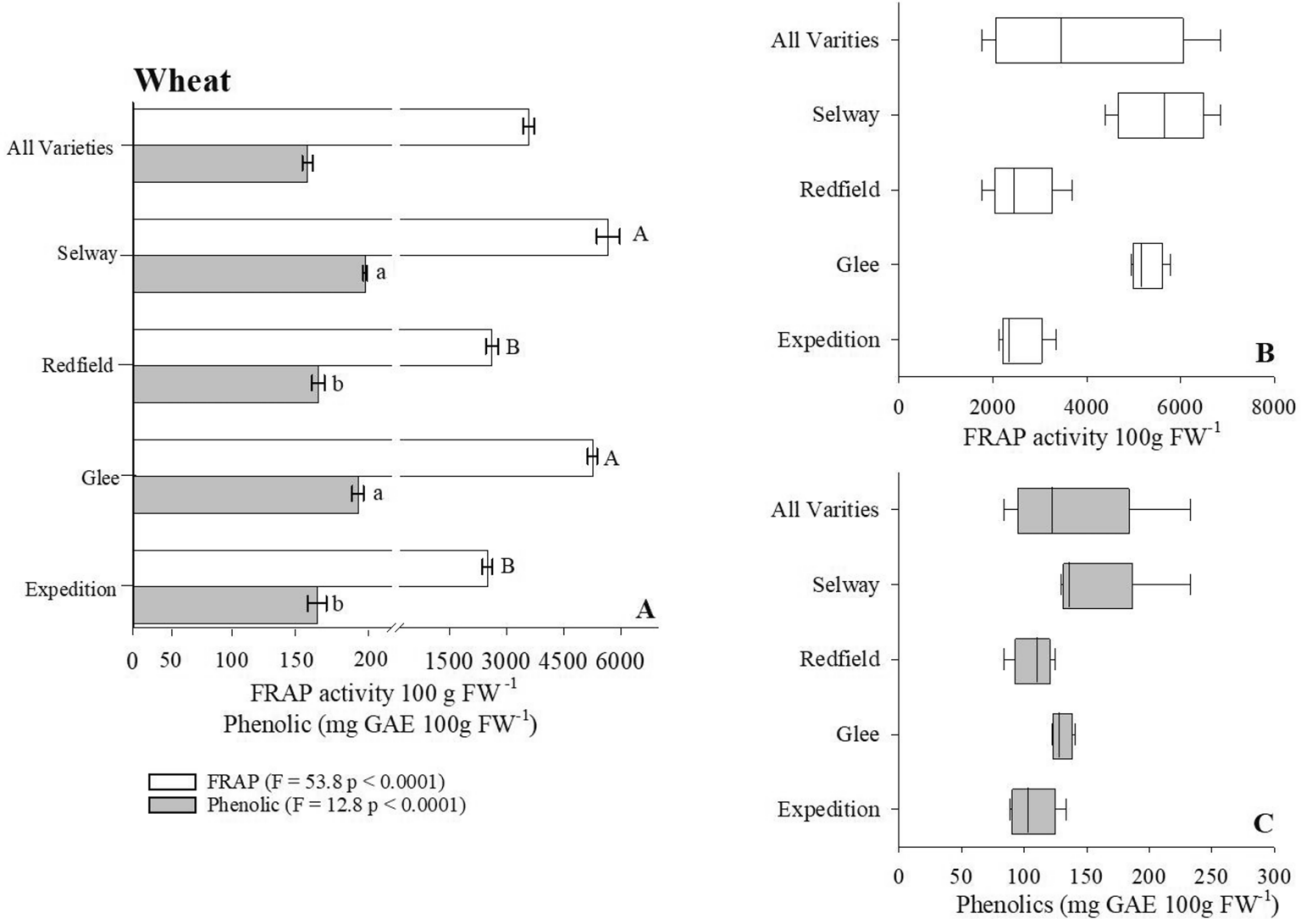
(**A**) Total ferric reducing antioxidant power (FRAP) activity and phenolic content for Wheat. ANOVA assessed variety differences for FRAP content within Wheat samples. Wheat phenolic assessment completed with ANOVA on log transformed data. Capital letters indicate significant differences between FRAP content, lowercase bold letters indicate significant differences between phenolic content (standard error bars with P < 0.05). (**A**) x-axis break occurs between 200 and 250. (**B**,**C**) represent Box whisker of FRAP and phenolic distributions. Box boundaries represent quartiles, the thick line in the box is the median and the whiskers represent the samples minimum and maximum. Wheat cultivars include Expedition (n = 9), Glee (n = 6), Redfield (n = 12), and Selway (n = 12).

We determined the antioxidant content (i.e., FRAP activity) of four crops commonly found in a wide range of diets using the Reflectometer as our evaluating platform. In the case of lettuce, our results indicate that Salanova Red and Red Oak cultivars on average contained twice the antioxidant content when compared to the remaining lettuce cultivars (Fig. 4A, Table 2). However, total antioxidant content varied extensively within the Salanova Red cultivar ranging 265–8600 (FRAP activity 100 g FW^−1^) with a general mean of 870 (FRAP activity 100 g FW^−1^ [Fig. 4B]). Assessing the antioxidant content of carrots, we observed that the Mokum cultivar maintained significantly greater concentration of antioxidants (*Χ*^2^ = 18.1, p < 0.001) when compared to remaining carrot cultivars (Fig. 5A, Table 2). The dramatic variation of antioxidant content was largest in Bolero (4.8–146, ${\overline{\text{x}}}$ = 35) and Mokum (9.4–161, ${\overline{\text{x}}}$ = 59 [Fig. 5B]) varieties. The antioxidant potential of oat and wheat cultivars was 2x − 10 × greater than the antioxidant potential measured in both lettuce and carrot cultivars (Figs. 6A, 7A, Table 3). Our results indicate that the oat cultivar Sumo contained significantly greater antioxidant capacity when compared to the three remaining cultivars evaluated (Fig. 6A, Table 3). The largest variation in antioxidant activity was observed in the oat cultivar Casino (2000–5500, ${\overline{\text{x}}}$ = 4200 [Fig. 6B]). Assessment of wheat cultivars we observed significantly greater antioxidant capacity in both Selway and Glee cultivars compared to remaining wheat varieties (Fig. 7A, Table 3). We observed limited variation in antioxidant capacity across all assessed wheat cultivars (Fig. 7B).

**Table 2:** Distribution of phytochemicals across lettuce and carrot cultivars.

Phytochemical		
Cultivar	Antioxidant activity 100 g FW^−1^	Polyphenols (mg GAE 100 g FW^−1^)
Lettuce		
Butterhead	966 (± 366)^ab^	18.0 (± 2.40)^c^
Green Romaine	239 (± 25)^c^	27.7 (± 1.60)^bc^
Muir	491 (± 90.0)^b^	54.0 (± 15.8)^ab^
Red Oak	2080 (± 533)^a^	95.0 (± 28.0)^a^
Salvanova Red	2080 (± 594)^a^	40.0 (± 5.70)^ab^
All varieties	652 (± 34.0)	76.0 (± 2.00)
^a^Romaine	*114 (*± *3.80)*	*63.5 (*± *3.50)*
^a^Red Oak	*665 (*± *22.3)*	*322 (*± *6.10)*
^b^Romaine varieties	*NR*	*27.4 (*± *4.20)*
^b^Red varieties	*NR*	*68.5 (*± *10.1)*
Carrots		
Bolero	44.3 (± 5.20)^b^	8.60 (± 0.60)^ab^
Mokum	67.1 (± 8.50)^a^	12.2 (± 1.70)^a^
Romance	30.0 (± 3.00)^c^	5.10 (± 0.98)^bc^
Rothid	44.0 (± 5.20)^b^	5.60 (± 0.47)^c^
Yaya	29.8 (± 6.00)^bc^	8.80 (± 1.40)^ab^
All varities	47.3 (+ 2.00)	7.40 (± 0.30)
^c^Orange variety	1.03* (NR)	14.5*(NR)

Values in the antioxidant activity and polyphenol columns with the same superscript letters are not significantly different (p < 0.05).

^a^Llorach et al.^39^.

^b^Liu et al.^57^.

^c^Singh et al.^61^.

NR indicates data Not Reported.

**Table 3:** Oat and wheat phytochemical distribution across cultivars.

Phytochemical			
Oats	Antioxidant activity 100 g FW^−1^	Polyphenols (mg GAE 100 g FW^−1^)	Protein (mg dry weight g^−1^)
Casino	3214 (± 202)^b^	128 (± 6.00)^a^	9.10 (± 0.52)^b^
Navaro	3076 (± 314)^b^	121 (± 7.30)^a^	8.30 (± 0.73)^b^
Ruffian	1587 (± 154)^c^	92.0 (± 6.60)^b^	12.4 (± 0.52)^a^
Sumo	4323 (± 216)^a^	135 (± 6.20)^a^	10.4 (± 0.47)^ab^
All varieties	3278 (± 170)	125 (± 4.20)	10.1 (± 0.30)
Wheat			
Expedition	2506 (± 131)^b^	106 (± 5.40)^b^	9.10 (± 0.53)^a^
Glee	5260 (± 130)^a^	130 (± 3.30)^a^	8.4 (± 0.71)^ab^
Redfield	2626 (± 162)^b^	107 (± 3.60)^b^	7.60 (± 0.51)^b^
Selway	5677 (± 299)^a^	134 (± 1.10)^a^	7.80 (± 0.40)^b^
All varieties	3591 (± 144)	101 (± 3.00)	9.00 (± 0.24)
*KanQueen*^+^	NR	*143 (*± *10.6)*	NR
* Cham1*^+^	NR	*180 (*± *6.20)*	NR
*Stoa*^+^	NR	*186 (*± *3.30)*	NR

Values in the antioxidant activity and polyphenol columns with the same superscript letters are not significantly different (p < 0.05).

^+^Okarter et al.^77^ polyphenol quantification reported as µmol GAE g DW^−1^ converted to mg GAE g- DW^−1^ for comparison.

#### Evaluating phenolic content across crop varieties

To determine the overall influence of cultivar selection on crop phenolic content we employed the Reflectometer in combination with the Folin Ciocalteau extraction methodology. Our initial assessment of lettuce cultivars suggests that Red Oak lettuce variety maintained the largest concentration of phenolic compounds (100 mg GAE 100 g FW^−1^), approximately 4 × greater than Butterhead lettuce sample (Fig. 4A, Table 2). As well, Red Oak cultivar had the greatest variation range (5–290, ${\overline{\text{x}}}$ = 20) in phenolic content across all analyzed samples (Fig. 4C). Turing our focus to carrot cultivars Bolero, Mokum and Yaya had significantly greater phenolic content when compared to remaining carrot varieties (Fig. 5A, Table 2). Variation in phenolic content was restricted in both the Bolero and Yaya cultivar however, we observed wide variation in the Mokum samples analyzed (5–37, ${\overline{\text{x}}}$ = 10 [Fig. 5C]). The phenolic content of oat cultivars suggest that the Ruffian variety contained the lowest measured phenolic content (125 mg GAE 100 g FW^−1^) compared to all varieties analyzed (Fig. 6C, Table 3). Additionally, both Navaro (43–160, ${\overline{\text{x}}}$ = 70) and Ruffian (43–150, ${\overline{\text{x}}}$ = 91) cultivars had the greatest variation in phenolic (Fig. 6C). In terms of wheat phenolic content, we observed a significantly greater concentration in both Selway and Glee cultivars (F = 12.8, p < 0.0001, ANOVA/log transformed) similar to our antioxidant observations (Fig. 7A, Table 3). We observed limited variation in phenolic content across all tested wheat cultivars (Fig. 7C).

#### Protein content of differing small grain varieties

**Figure 8:**
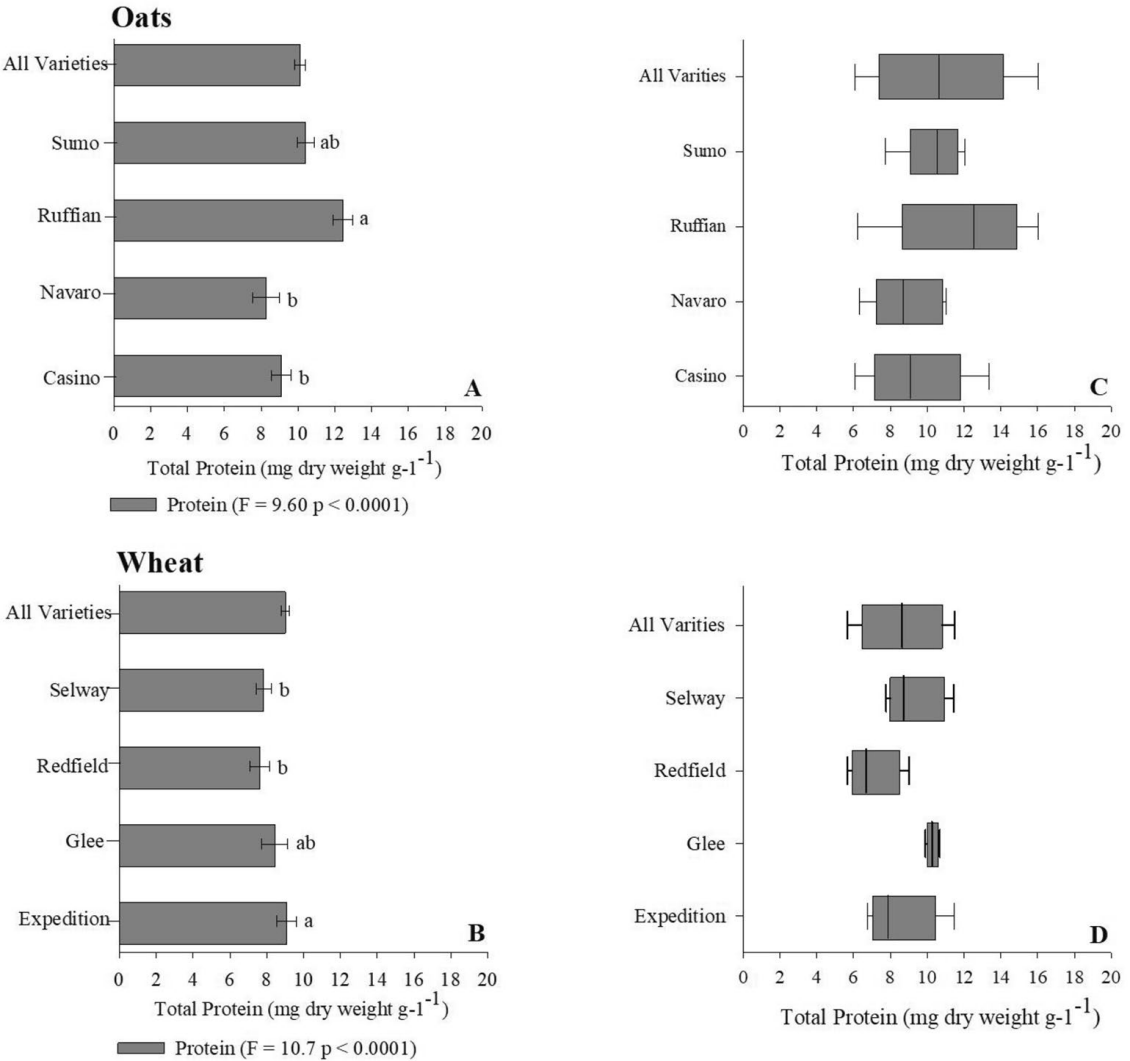
(**A**,**B**) Total Protein for Oat and Wheat assessment completed with ANOVA on log transformed data. Differences in lowercase letters indicate significant differences in total protein content (standard error bars with P < 0.05). (**C**,**D**) represent Box whisker of total protein distributions. Box boundaries represent quartiles, the thick line in the box is the median and the whiskers represent the samples minimum.

We evaluated small grain (i.e., oat and wheat) protein content across multiple cultivars using the Lowry protein extraction protocol in combination with the Reflectometer. Our preliminary review of protein content (mg dry weight g^−1^) of oat varieties indicates that Ruffian cultivar had significantly greater (F = 9.60, p < 0.0001, ANOVA/log transformed) protein than both Navaro and Casino cultivars (Fig. 8A, Table 3). This contrasts with both antioxidant activity and phenolic content observations where both Navaro and Casino maintained significantly greater concentrations when compared to Ruffian (Fig. 6A, Table 3). In terms of protein concentration variability Ruffian cultivars had the greatest variation (6.2–16, ${\overline{\text{x}}}$ = 12.5 [Fig. 8C]). Results from our assessment of wheat cultivars mirror that of our oat findings, the cultivar with the lowest antioxidant capacity and phenolic content (i.e., Expedition) has significantly greater protein concentrations (F = 10.7, p < 0.0001, ANOVA/log transformed) when compared to remaining wheat varieties (Fig. 8B, Table 3). Following that line of observations, the Redfield wheat cultivar was lowest across all phytochemicals assessed (Fig. 7A, 8B, Table 3). Similar to previous findings we observed limited variation in protein content across all tested wheat cultivars (Fig. 8D).

## Discussion

### Methodology validation study

There is a significant need to facilitate large scale observational studies investigating the multifaceted linkages between farm management, soil health, and post-harvest/supply chain impacts on crop nutrient quality. Dwivedi et. al.,^11^ suggest that the compulsion to increase crop yield has led to an overall loss in crop nutrient quality, they base their hypothesis on two landmark studies from the UK^6^ and US^17^ demonstrating marked reductions in key nutrients across several decades. The authors further suggest that evaluating the connections between crop production and nutrient quality has gone largely under investigated due to substantial analytical costs associated with assessing crop nutritional status. Analytical costs are not limited to instrumentation but also by the complexity of data collection as well as processing. Using this understanding as our backdrop we evaluated the Reflectometer capacity to accurately assess phytochemical nutrient quality of crop samples against a commercial laboratory grade spectrometer. Reflectometers have proven to be a robust analytical tool assessing soil-C content. Specifically, Ewing et al.^18^ observed that the Our Sci reflectometer precisely assessed soil-C concentrations with sufficient accuracy to provide critical information regarding management decisions. Herts et al.^19^ further suggest that the Our Sci reflectometer proved effective at determining SOC content based on captured spectral data.

Numerous definitions have been developed to describe antioxidants^20^, broadly speaking antioxidants are molecules capable of scavenging and/or inhibiting the production of free radicals (e.g., highly unstable atoms, molecules, or ions with unpaired electrons) consisting of reactive oxygen species (ROS), reactive nitrogen species (RNS), or non-free-radical species^21^. Colorimetric assays are the method of choice to assess antioxidant potential of specific solutions typically relying on two possible mechanisms hydrogen atom transfer (HAT) or single electron transfer (SET) inducing a color change. In the case of the FRAP assay single electron transfers occurs when ferric ions (Fe^3+^) are reduced to ferrous ions (Fe^2+^) by sample antioxidants suspended in acidic media (pH < 3.6) releasing blue color indicative of the Fe^2+^-TPTZ complex^22^. The absorbance peak for the Fe^2+^-TPTZ is 593 nm studies suggest this is the ideal wavelength however, absorbance reading spanning a range of 560–620 nm have been shown to provide valid results^23^. In the case of our study spectra data from the FRAP reaction collected by the StellarNet spectrometer spanning a wavelength of 540–600 nm suggest undetectable to slight variation within absorbance data (Fig. 1A). Comparison of StellarNet spectrometer absorbance and Reflectometer data suggest a strong correlation (r^2^ > 0.90) in the case of both carrot and lettuce samples (Fig. 1C). This observation provides support that the Reflectometer is an applicable analytical tool for the assessment of antioxidants in plant samples via FRAP assay. Finally, we noted that the Reflectometer underestimated the antioxidant potential of both carrot and lettuce when compared to StellarNet spectrometer (Fig. 1D). A noted limitation with the FRAP assay suggests that compounds within a sample can result in interference due to UV–VIS adsorption at wavelengths of 593 nm resulting in an overestimation of FRAP potential^23,24^. Based on this observation it is possible the Reflectometer provided greater accurate accounting of FRAP content in both carrot and lettuce samples.

Phenolics represent important ubiquitous compounds in the plant kingdom^25^, structural chemistry consists of possessing one or more benzene ring bearing one (phenol) or more (polyphenol) hydroxyl substituents and are generally categorized as phenolic acids, flavonoids, stilbenes, coumarins, and tannins^5,26^. Phenolics function as defense/protection compounds limiting herbivory by alternating tissue palatability^27^, hindering microbial infection^28,29^ as well as protecting plants from ultraviolet radiation and resulting oxidant damage^30^. Total phenolic content of plants is typically evaluated using the Folin-Ciocalteu assay (FC-assay^31^). Singleton and Rossi^32^ improved the FC-assay to specifically measure phenolic concentration by shifting the reducing agent from molybdotungs to-phosphoric heteropolyanion, adjusting the spectrometer wavelength. During the reaction the FC reagent oxidizes phenolic compounds resulting in a blue colored reduced FC reagent, which is measured at 760 nm with color intensity correlating with phenolic content of the sample. As expected, both the StellarNet spectrometer (Fig. 2A) and reflectometer (Fig. 2B) provided strong linear relationship (r^2^ > 0.9) evaluating phenolic content as concentrations of GAE standard curve increased. Additionally, we observed robust linear agreement (r^2^ > 0.9) between StellarNet spectrometer and the Reflectometer data for lettuce samples (Fig. 2C). However, liner fit comparing StellarNet spectrometer, and Reflectometer provided very good agreement for carrot samples (r^2^ > 0.8, Fig. 2C). Previous studies utilizing reflectometers in combination with test strips to assess soil nitrogen and phosphorus availability note that limitations in the accuracy of reflectometers can occur for numerous reasons including chemical interferences, spectra restrictions, and out of range nutrient densities^14^. Based on this understanding we would suggest that the Reflectometer provides a valid assessment of phenolic content in carrot samples potentially underestimating phenolic potential (Fig. 2D) however, additional fine-tuning of the reflectometer could improve detection efficiency.

Plant storage proteins consist of biological macromolecules facilitating plants in meeting enzymatic, growth, and nutritional demands^33^. Plant proteins can be classified as either seed storage proteins ([SSP] i.e., cereal grains, legumes, oil-seed legumes, and nuts) or vegetative storage proteins ([VSP] i.e., tubers), given that our analysis focuses on protein content of grains in both oat and wheat cultivars, we will concentrate our discussion on SSP. Fundamentally SSP’s are plant mediated amino acid reservoirs, where stored proteins are safely sequestered from degradation until activated by seed germination ques^34^. Several colorimetric methods have been developed to evaluate SSP concentrations, common methods include Biuret method, Lowry method, Bradford assay, and the Bicinchoninic acid (BCA) assay^35^. The described methods rely on either metal ion (Biuret method), dye binding (Bradford assay) or a combination of both (Lowry method and BCA assay) to initiate a color change reaction detectable via spectroscopy range of 650–750 nm^35^. In the case of our study, we utilized the Lowry method to assess protein content in oat and wheat grain samples, both the StellarNet spectrometer and the Reflectometer provided a strong linear fit (r^2^ > 0.9) with increasing concentrations of BSA standard curve (Fig. 3A,B). However, comparison of the StellarNet spectrometer and Reflectometer assessment of oat and wheat protein content provided a modest linear fit (r^2^ > 0.8) suggesting a less than ideal agreement between detection methods (Fig. 3C). Oat and wheat samples analyzed by the StellarNet spectrometer, and Reflectometer were subjected to the same extraction process suggesting that the discrepancy in linear fit is likely due to measured spectra differences between each analytical tool rather than limitations associated with extraction. It is well documented that phenolic compounds can significantly interfere with spectra data generated from the Lowry method^36^. In the case of our oat and wheat samples phenolic concentrations outpace protein content at a ratio of 10:1 (Figs. 6, 7, 8, Table 3), it is possible that increased phenolic content of our small grain samples interfered with resulting spectra data. Potential follow up studies should include validating the Reflectometer results against Kjeldahl’s method which quantifies amino acid in proteins is not prone to interference bias and/or investigating extraction buffers that reduce phenolic content potentially providing improved linear agreement.

Overall, the Reflectometer provided very good estimate of most phytochemicals assessed across crop samples with the except for small grain proteins. Although, the linear fit was less then optimum (r^2^ > 0.8) the Reflectometer provided a reasonable estimate of both phytochemicals. Finally, repeatability assessment demonstrated good reproducibility of the Reflectometer to assess crop phytochemical content, Mallstellone et al.^37^ suggests that RSD % values lower than 15% indicate good reproducibility of proposed methods. There is a growing trend to develop inexpensive, accessible, analytically robust spectrophotometers and reflectometers capable of accurately assessing extracted plant-based phytochemicals^38^, our observations suggest that the Reflectometer exceeds these required characteristics.

### Crop phytochemical variability study

Agronomic research efforts have assessed specific influences on crop phytochemical profiles including variety selection^39,40^, farm management^41–43^, nutrient availability^44,45^, as well as insect and pathogen pressure^46,47^. As part of our investigation, we collected crop samples from wide range of management practices, soil types and environmental conditions, assessing the antioxidant, phenolic, and protein content of specific cultivars (i.e., lettuce, carrots, and small grains). Our study suggests there is a role for crop variety in driving phytochemical accumulation but identifies variations within specific cultivars that suggest it is far from a dominant factor. There exists a good understanding of individual factor effects on specific crop phytochemical content, however, there is a critical need to expand our knowledge taking an integrated approach to evaluating the interactions of all contributing components.

A growing body of research suggests that crops elevated in phytochemicals such as antioxidant and phenolic compounds are a vital dietary component improving human health outcomes potentially reducing the risk of numerous debilitating diseases including diabetes, cardiovascular disease, and obesity^48–50^. The function of antioxidants involves scavenging cellular concentrations of free radicals (i.e., reactive oxygen species), in the case of plants free radicals occur because of metabolic function and environmental stress events including high light intensity, temperature fluctuations, drought, and pathogen/herbivory^51^. Phenolic compounds in plants serve multiple functions including acting as antioxidants, metal chelators, defense/protection compounds limiting herbivory^52^, hindering microbial infection^53^ as well as protecting plants from ultraviolet radiation and resulting oxidant damage^54^.

Lettuce contains several bioactive phytochemicals essential to human health including polyphenols, carotenoids, and vitamin C^39,55^. Per capita consumption of lettuce in the United States has been steadily increasing over the last several decades^56^ potentially due to its year-round availability and minimal need for extensive processing. Provided the importance of lettuce as a dietary staple and its phytochemical potential studies are beginning to evaluate the antioxidant and phenolic content of several lettuce genotypes. Our results suggest that Salvanova Red and Red Oak lettuce varieties had a two-fold increase in antioxidant content compared to both Muir and Green Romaine varieties; measurement of phenolic compounds suggest a similar trend (Fig. 4A, Table 2). This observation is in line with findings of previous studies, Llorach et al.^39^ found that red lettuce varieties maintained significantly greater FRAP assessed antioxidant potential 2–8 × greater than green lettuce varieties (Table 2). However, their antioxidant potential (FRAP) for red variety lettuce was 3 × less compared to our results yet 3 × greater in phenolic content (Table 2), differences in results are potentially the result of sample preparation (i.e., free-drying vs fresh ground samples) and phenolic evaluation methods (i.e., HPLC vs. Colorimetric). Similarly, Liu et. al.,^57^ utilizing detection methodologies comparable to our approach observed that red pigmented lettuce cultivars propagated under identical conditions had greater antioxidant and phenolic capacity compared to green cultivars (Table 2), additionally results further suggest that red lettuce harvested in July maintained greater antioxidant potential and phenolic content then September harvested crops indicating a link between seasonal effects and antioxidant accumulation. Knowledge of genotype differences in phytochemical accumulation especially in lettuce has encouraged additional investigations into both environmental and management factors influencing antioxidant content. For example, Yang et al.^58^ observed that lettuce cultivars fertilized with glycine (N-source) accumulated greater concentrations of phenolic compounds and maintained greater antioxidant activity then control treatments not amended with glycine.

Carrots are a significant economically viable crop with estimated annual production exceeding 37 million tons^59^, the predominant variety is typically orange-colored^60^. The orange carrot cultivar is the result of a chance mutation originating in the Netherlands circa the seventeenth century, due to its shape, crop uniformity, smooth surface texture and bolting tolerance the orange carrot became poplar globally^61^. Using this understanding, we evaluated the antioxidant potential and phenolic content of orange colored carrot varieties. Our observations suggest that orange carrots regardless of cultivar recorded the lowest antioxidant content of all crops evaluated, with approximately 10 to 40 times less compared to both lettuce and small grains respectively (Figs. 3A, 5A, Table 2). We observed that of the varieties assessed Mokum maintained the greatest antioxidant/phenolic content followed by Bolero, Romance, Yaya and Rothlid (Fig. 5A, Table 2). Multiple studies have evaluated antioxidant and phenolic potential comparing orange cultivars to black, purple, red and yellow, varieties, in all instances’ colored genotypes have 10–100-fold greater antioxidant content than orange genotypes^62–65^. The primary phytochemicals in orange carrots are β-carotene and α-carotene, compounds critical for both vision and immune system health^66^. Singh et al.^61^ reported antioxidant activity (FRAP) 40–60 × less than our observed values however, phenolic content (Folin Ciocalteau method) was within range of our results (Table 2). Further they observed a significant negative correlation between carotene, antioxidant activity and total phenolic concentration^61^, Koley et al.^67^ observed a similar negative correlation between β-carotene, FRAP activity and total phenolic content in Indian carrot cultivars.

The phytochemical content of small grains was initially considered inconsequential when compared to both fruits and vegetables, this paradigm shifted considerably once advanced extraction protocols were developed releasing bound phytochemicals within small grains^68^. This improved understanding suggests that whole grains, especially corn, oats and wheat maintain greater antioxidant and phenolic content compared to most fruits and vegetables^69^. Oat and wheat samples as part of this study underwent lengthy extraction process (i.e., methanol and sodium hydroxide) releasing both free and bound phytochemicals^70^. Our results indicate that both oat and wheat samples contain 30-fold greater antioxidant activity compared to lettuce samples; phenolic concentrations remain equivalent between both crop types (Figs. 4A, 6A, 7A, Tables 2, 3). Additionally, we observed significant differences in both antioxidant activity and phenolic content between varieties of oats and wheat samples (Figs. 6A, 7A, Table 3). There exists significant difficulty in drawing direct comparisons between studies assessing the phytochemical content of small grains and results collected as part our study especially in terms of oat phytochemical content. Specifically, the multitude of antioxidant detection methodologies (i.e., ABAP, 2,2-azobis amidinopropane, DPPH 2,2-diphenyl-1-Picrylhydrazyl, TOSC, total oxyradical scavenging capacity), extraction protocols/solvents utilized and reporting of units assessing antioxidant activity (i.e., percentage, Trolox, Fe equivalents). Clarke et al.^71^ suggested there is general redundancy between antioxidant detection methods however, differences in extraction methods may result in underestimation of small grain phenolic content^72^. Nonetheless, several studies have observed clear differences in oat antioxidant activity^73^ and phenolic^74,75^ content suggesting the capacity of specific oat genotypes to influence phytochemical accumulation. Similar findings have been suggested in terms of wheat phytochemical accumulation, Verma et al.^76^ observed significant differences in wheat phenolic content and antioxidant activity across 51 wheat genotypes propagated during the same season and geographical region. Okarter et al.^77^ evaluated six-wheat varieties noting significant differences in phenolic content and antioxidant activity resulting from genotype. Furthermore, the phenolic concentrations identified by^77^ were within range of our results (Table 3). In contrast Adom et al.^70^ observed no difference in antioxidant activity and only moderate differences in phenolic content of 11 wheat cultivars. Their results further suggest that the majority of wheat total phenolic content remains in the bound fraction. This finding has significant health implications as suggested by Anderson et al.^78^, they hypothesize that bound phenolics survive early digestion and are absorbed later in the lower gastrointestinal tract providing resistance to several chronic diseases. Provided the understanding that genotype is a significant influence effecting crop phytochemical accumulation, studies have begun to assess additional controlling factors including environment and management. For example, Zuchowski et al.^79^ investigated variations in spring and winter wheat phenolic content due to differing management (i.e., organic vs conventional) practices. Their results suggest that organic managed winter wheat maintained greater concentrations of specific phenolic compounds, they attributed their findings to possible deficiencies in soil-N and genotype kernel morphology. Current understanding of specific mechanisms controlling phytochemical accumulation remains in its early stages additional studies are needed to fully understand the effect of environment, genotype, and management.

Protein is a significant constituent of all small grains and is subject to variation resulting from environment, genotype, and management changes. However, protein concentrations are typically greater in oat and wheat compared to other small grains^80^. Specifically, oat and wheat protein content are highly susceptible to both environmental pressure and management decisions^74^. Ryan et al.^81^ observed greater wheat protein synthesis in crop rotations that incorporated either legumes or greater N-fertilization suggesting the link between protein and N-availability. We observed significant variation in protein accumulation as a result of genotype differences in both oat and wheat samples (Fig. 8A,B, Table 3). Additionally, within both genotypes that reported the largest protein concentrations maintained the lowest antioxidant activity and phenolic content (i.e., Ruffian and Expedition [Figs. 5A, 6A, Table 3]), suggesting a tradeoff between phenolic content and protein biosynthesis. Tong et al.^82^ suggested that oat protein accumulation could be used as a predictor of phenolic content due positive linear correlation (r^2^ = 0.66, p < 0.01) observed between phytochemicals, in contrast our result suggest a less robust negative linear fit between oat protein and phenolic content (r^2^ = 0.35, p < 0.0001: data not provided). In terms of wheat our results indicate a positive modest linear relationship between protein and phenolic content (r^2^ = 0.22, p < 0.002: data not provided), Pasqualone et al.^83^ observed a significant linear relationship between wheat grain protein content and total soluble phenolic compounds. To the authors knowledge studies assessing the biochemical relationships between small grain protein accumulation and phenolic content have not been fully realized.

In general, several factors govern accumulation of phytochemicals including cultivar genotype, environment, and management practices. Despite this understanding disentangling the overall effect of specific conditions on phytochemical biosynthesis at the field level is extremely complex requiring accounting crop variety characteristics, and management inputs across multiple seasons. Results collected from our study demonstrate the significant influence of crop variety on phytochemical variation. Further, we noted large variation in antioxidant activity and consequently phenolic content within individual cultivars of lettuce (i.e., Salvanova Red and Red Oak [Fig. 4B,C]), carrots (i.e., Bolero and Mokum [Fig. 5B,C]), oat phenolic content (Ruffian and Navaro [Fig. 6A, Table 3]) and to a lesser extent oat antioxidant capacity (Casino [Fig. 6B]). Based on our data we hypothesize that hyperproducers of phytochemicals such as the case of Salvanova Red lettuce are extremely sensitive to both environmental fluctuations and management inputs. Furthermore, crop samples submitted for analysis as part of this study were collected across numerous management practices and environments, it is possible that the extensive variation we observed was the result of specific conditions overwhelming the capacity of the hyperaccumulator crop to biosynthesize phytochemicals. Future studies should evaluate the influence of environment and crop management on phytochemical accumulation spanning several crop types (i.e., fruits, vegetables, and small grains) and cultivars. Deciphering how crop variety, environment, and management influence crop phytochemical biosynthesis would provide critical insight needed to improve overall crop nutrient quality through management practices. This assessment will be progressively vital as the incidences of diet related illnesses continue to increase.

## Methods

### Methodology validation study

#### Preparation of standard curves

All methods were carried out in accordance with relevant institutional guidelines and regulations. Testing the capacity of the Reflectometer to evaluate phytochemical content initially involved generating standard curves for each phytochemical of interest. The standard curve for the ferric reducing antioxidant power (FRAP) assay was prepared by dissolving 0.1 g of ferric chloride (MPBIO) into 10 mL of deionized water creating a 62 × 10^4^ µM stock solution. Next the ferric chloride (FeCl^3^) stock solution was diluted at a ratio of 1 part stock solution to 35 parts deionized water. The final diluted solution was distributed into cuvettes at concentrations ranging from 0 to 200 µM FeCl^3^, FRAP activity is expressed in µmol Fe^2^, in crop samples expressed as FRAP activity 100 g FW^−1^. Total phenolic content was measured using the Folin Ciocalteau method which employs dilutions of gallic acid to produce a standard curve. Briefly, standard stock solution was prepared by mixing 0.1 g Gallic acid (Sigma-Aldrich) with 100 mL of Methanol (Pharmco). Next the stock solution is diluted with deionized water generating a standard curve ranging from 12.5 to 200 μg mL^−1^, total phenolic content is expressed as gallic acid equivalent (mg GAE g sample DW^−1^) in crop samples. The standard curve for the Lowry method (total protein) was developed by mixing 100 µL of Bovine Serum Albumin (BSA) standard solution (Biobasic) into 900 µL of deionized water. BSA stock solution was diluted producing a standard curve ranging from 0 to 500 µg BSA mL^−1^, total protein content in crop samples is expressed as mg dry weight g^−1^.

#### StellarNet spectrometer

Commercial spectroscopic measurements were carried out with the StellarNet BLUE-Wave UV–visible Spectrometer (StellarNet Inc., Tampa, FL, USA) in combination with Ocean Optics DH-2000-Bal serving as a light source. The StellarNet BLUE-Wave Spectrometer is a fiber-optic-coupled instrument for measurements in the range of 350–1150 nm wavelength. For the purposes of our study light transmittance, reflectance, and calculated absorbance was analyzed from 540 to 600 nm antioxidant (FRAP assay), 720–770 nm phenolic (Folin Ciocalteau method) and 745–755 nm total protein (Lowry method).

#### Reflectometer

Antioxidant, phenolic, total protein standard curve as well as crop phytochemical reflectance spectra were collected using the Reflectometer (Our Sci, LLC; http://our-sci.net). Ewing et al.^18^ provides a complete description of the reflectometer. Briefly the tool hardware is licensed under the GNU General Public License v3.0. The reflectometer includes 10 light emitting diodes ranging from UV-B to near-infrared wavelengths (365, 385, 450, 500, 530, 587, 632, 850, 880, and 940 nm). During measurement phase light sources are pulsed, allowing software to filter ambient light, and reflection is measured using two pin photodiode detectors in the 300–700- and 700–1000-nm ranges. Reflectometer can be used in combination with an Android™ phone or tablet. Reflectometer connection to SurveyStack via Bluetooth, is an advanced open-source web application (https://app.surveystack.io).

#### Repeatability assessment

Reflectometer precision was evaluated by performing Relative Standard Deviation (RSD%) assessment. Standardized samples (n ~ 5) representing fruit and vegetable (i.e., Butternut squash) and grain (i.e., Wheat) were extracted utilizing described methods for antioxidants (FRAP), polyphenols (Folin Ciocalteau method) and protein (Lowry method [Wheat only]). Four inter-day sampling evaluations were completed for each phytochemical of interest apart from grain antioxidant and protein content (3-sampling periods [Table 1]).

#### SurveyStack and SurveyStack Kit

SurveyStack progressive web application was built using NodeJS/express with mongoDB on the Server and Vue with Vuetify on the Client. Hardware integration between SurveyStack and the Reflectometer occurs via the SurveyStack Kit mobile application written in Kotlin for Android devices. The source code for all applications is available and documented on Gitlab (https://gitlab.com/our-sci/software/surveystack). The Android application is available for download over the Google Play store (https://play.google.com/store). SurveyStack and SurveyStack Kit are licensed under the GNU General Public License v3.0.

All data collection was completed using SurveyStack forms. For each data collection activity, forms were created to guide users through each aspect of the protocol, including Reflectometer measurements, instructions, and questions for entering metadata (ex: crop type, amount of extractant used, etc.). An automated data pipeline was built using SurveyStacks API’s to merge data from each completed survey and mongoDB scripts (https://gitlab.com/our-sci/real-food-campaign/lab-data-review-dashboard/-/tree/main) calculated measurement outcomes.

### Crop phytochemical variability study

#### Crop sample characteristics

Crop samples were submitted from both producer and consumer volunteers from 2019 to 2022 representing 10,000 unique samples with accompanying geographical and management data where applicable. As part of our study, we selected 306 samples representing four crops including leafy greens (lettuce) root crops (carrots) and small grains (oats and wheat). Selection criteria included n > 5, complete set of phytochemical data, as well as associated geographical and management information.

#### Sample storage, and preparation

All submitted samples were stored at ~ 4 °C before processing. Samples were initially inspected assessing overall quality (significantly decomposed samples were not utilized), further measurements include sample weight, density, and moisture content. Carrot and lettuce samples were thoroughly washed with water, in the case of carrot samples, skins were removed prior to processing. Lettuce samples were ground to a fine paste using a commercially available coffee grinder. Carrot samples were grated using a Cuisinart boxed grater from the coarse shredding size. Sample preparation was kept at a minimum (< 2–3 min) to minimize sample oxidization. In the case of both oat and wheat a representative homogenized subsample (5–8 g) was initially assessed for moisture content by collecting a primary weight then drying sample for 24 h. at 70 °C and reweighing sample. The sample was then ground with a commercially available coffee grinder for 30 s, sieved at 0.5 mm, and stored at 0 °C.

#### Sample extraction for phytochemical assessment

80% methanol (Pharmco) was added to each sample extract and sonicated for 1.0 h. at 25 °C. Sample weight to the 80% methanol ratio for lettuce samples were 2 g : 8 mL, for carrot, 5 g : 5 mL, for wheat 1 g : 8 mL and for oat 1 g : 10 mL. In the case of dark purple carrots, the sample weight was 1 g: 5 mL. After sonication samples were placed on shaker table (150 rpm) for an additional 1.0 h. At the conclusion of incubation process all samples were centrifuged at 3000 rpm for 15 min. Supernatant was collected in clean tube and stored at 0 °C until FRAP, Phenolic, or Total Protein evaluation.

#### Antioxidant assessment: FRAP assay

To evaluate the antioxidant activity of crop samples via iron reduction, we employed the ferric reducing antioxidant power (FRAP) assay, as described by Benzie and Strain (1999) with minor modifications. Briefly the FRAP reagent was prepared immediately prior to sample analysis by mixing 24 mL of acetate buffer (300 mM, pH 3.6, [Bio-techne]), 3.0 mL of TPTZ (tripyridyltriazine, [CHEM IMPEX]) solution (10 mM TPTZ in 40 mM HCl [CHEM IPEX]), and 3.0 mL of FeCl^3^ (20 mM [MPBIO]) in aqueous solution. An aliquot of 500 µL of sample extract was added 500 µL of FRAP reagent vortex for 30 s and incubate at 20 °C for 10 min. Sample absorbance was measured at 587 nM with the Reflectometer. Standard curve was constructed using ferrous chloride (0–200 μM) and the results were expressed in µmol Fe^2+^ equivalents.

#### Determination of total phenolic content: Folin Ciocalteau method

Total phenolic content analysis was assessed using the Folin Ciocalteau spectrophotometric method described by Singleton et al.^84^. Crop extracts were extracted in methanol 80% (as described above), 200 µL of crop sample was transferred to a tube with 3.5 mL of deionized water, 100 µL of Folin Ciocalteau reagent (Sigma-Aldrich) and 200 µL of 20% sodium carbonate (Biobasic). The mixture was vortexed for 30 s, then secured to a shaker table and mixed for 1.5 h. 150 rpm, samples were kept in the dark during mixing. Afterwards, the absorbance was measured at 850 nm using the Reflectometer. A blank test was also performed under the same conditions and the results of total phenolic compounds were expressed as gallic acid equivalent (mg GAE g sample DW^−1^), based on a calibration curve of gallic acid in the concentration range of 0 to 200 μg mL^−1^.

#### Total protein: Lowry method

Oat and wheat grain samples were initially extracted with 80% methanol (as described above). After centrifugation methanol is decanted and the remaining pellet is resuspended in 50 mL of 0.1 M Sodium Hydroxide (Biobasic) placed on a shaker table for 2.0 h. at 150 rpm. Upon completion Sodium Hydroxide extraction step samples were centrifuged at 3000 rpm for 15 min. 100 μL of sample supernatant is suspended in 100 μL of deionized water. Total protein content for oat and wheat samples was assessed using the Biobasic Lowry Protein Assay Kit following manufacturer’s instructions. Standard curve was prepared by mixing 100 µL of BSA standard solution (Biobasic) into 900 µL of deionized water. BSA stock solution was diluted producing a standard curve ranging from 0 to 500 µg BSA mL^−1^, total protein content in crop samples is expressed as mg dry weight g^−1^.

### Statistical analysis

In the methodology validation study, we used linear regression to test for correspondence between commercial spectrometer absorbance spectra vs. phytochemical standard curves (SigmaPlot 14.5 2020, SYSTAT). Assessment of the Reflectometer utilized a 2-order polynomial regression model due to the diffuse reflectance spectroscopy produced by the Reflectometer. Briefly, the nature of the Reflectometer lends to a non-linear response, data accuracy is improved through use of non-linear calibration curves as opposed to transformations to linearize collected data (SigmaPlot 14.5 2020, SYSTAT). Finally, linear regression analysis was used to assess correspondence between the Reflectometer reflectance data from crop phytochemical concentrations vs. commercial spectrometer absorbance spectra crop phytochemical concentrations (SigmaPlot 14.5 2020, SYSTAT).

Data normality and homogeneity were reviewed for spectrometer absorbance, reflectometer reflectance spectra, and phytochemical data prior to analysis of variance (ANOVA) review. Oat phenolic, wheat antioxidant and wheat phenolic data were log transformed to meet required assumptions for ANOVA testing. ANOVA was used to test differences in spectrometer absorbance vs reflectometer reflectance spectra, and phytochemical concentrations within crop cultivars. Where F-ratios were significant (probability (p) [p < 0.05]) treatment means were compared via Tukey–Kramer multiple comparison tests (JMP, SAS Institute, Cary, NC, USA). Non-parametric analyses (Kruskal–Wallis test) were used if data failed to meet parametric assumptions. Where H-values were significant (p < 0.05) treatment means were compared via Kruskal–Wallis Wilcoxon One way Analysis test (JMP, SAS Institute, Cary, NC, USA).

## Data Availability

All data derived from the Bionutrient Institute methods are available publicly from our repository: https://gitlab.com/our-sci/bionutrient-institute/dataset. The data used in this manuscript covers samples submitted up to 7/31/2022. Our Sci seeks to increase transparency and access to research via open-source hardware and software and open-access data^19^.
